# Intravesical ALT-803 and BCG Treatment Reduces Tumor Burden in a Carcinogen Induced Bladder Cancer Rat Model; a Role for Cytokine Production and NK Cell Expansion

**DOI:** 10.1371/journal.pone.0096705

**Published:** 2014-06-04

**Authors:** Evan Gomes-Giacoia, Makito Miyake, Steve Goodison, Aravindhan Sriharan, Ge Zhang, Lijing You, Jack O. Egan, Peter R. Rhode, Alexander S. Parker, Karl X. Chai, Hing C. Wong, Charles J. Rosser

**Affiliations:** 1 Cancer Research Institute, MD Anderson Cancer Center Orlando, Orlando, Florida, United States of America; 2 Department of Health Sciences Research, Mayo Clinic, Jacksonville, Florida, United States of America; 3 Department of Pathology, MD Anderson Cancer Center Orlando, Orlando, Florida, United States of America; 4 Altor Bioscience Corporation, Miramar, Florida, United States of America; 5 Burnett College of Biomedical Sciences, University of Central Florida, Orlando, Florida, United States of America; 6 Clinical and Translational Research, University of Hawaii Cancer Center, Honolulu, Hawaii, United States of America; Oklahoma University Health Sciences Center, United States of America

## Abstract

Intravesical Bacillus Calmette-Guérin (BCG) has been shown to induce a specific immunologic response (*i.e.*, activation of IL-2 and effector T-cells), while preclinical studies using ALT-803 (mutated IL-15 analogue combined with IL-15Rα-Fc fusion) have shown promising results by prolonging the agent's half-life and stimulating CD8+ T-cells. Based on these results, we hypothesized that the intravesical administration of ALT-803 along with BCG will generate an immunologic response leading to significant bladder tumor burden reduction. Using a well-established carcinogen induced rat non-muscle invasive bladder cancer (NMIBC) model, we studied the effects of intravesical ALT-803 with and without BCG. Rat tissues were evaluated to document treatment response. Intravesical ALT-803 was safe and well tolerated alone and in combination with BCG. As a single treatment agent, ALT-803 reduced tumor burden by 35% compared to control whereas BCG alone only reduced tumor burden by 15%. However, the combination of ALT-803 plus BCG reduced tumor burden by 46% compared to control. Immune monitoring suggested that the antitumor response was linked to the production and secretion of IL-1α, IL-1β and RANTES, which in turn, induced the proliferation and activation of NK cells. Lastly, tumoral responses of the combinational treatment were associated with 76% reduction in angiogenesis, which is significantly higher than when assessed with either agent alone. The enhanced therapeutic index seen with this duplet provides justification for the development of this regimen for future clinical trials.

## Introduction

Bladder cancer (BCa) is the fifth most common malignancy in the United States, to be diagnosed in approximately 74,690 patients resulting in approximately 15,580 deaths in 2014 [1]. Seventy to eighty percent of patients with BCa present with non-muscle invasive bladder cancer (NMIBC), which is cancer confined to the mucosa of the bladder [2], [3]. NMIBC is treated by transurethral resection of the tumors followed by the administration of adjuvant intravesical chemotherapy or intravesical Bacillus Calmette-Guérin (BCG), which can decrease recurrence rates and prolong the progression-free interval [2]–[4]. Despite this, approximately 50–60% of NMIBC patients who undergo resection and intravesical therapy will experience a tumor recurrence [5]. Moreover, of those who experience a recurrence, approximately 30% will progress and succumb to their disease over a 15-year period, while another 50% will undergo radical cystectomy in an attempt to control their disease [6]. As such, a key issue in the field remains the need for better therapeutic alternatives to reduce recurrence rates, improve survival rates and obviate the need for radical surgery.

The exact mechanism of action by which intravesical BCG exerts its anti-tumorigenic effects is not known. However, it is clear that BCG activity is dependent on induction of inflammatory responses involving activation of multiple types of immune cells. Initially, intravesical BCG binds to the urothelial lining and is then internalized and processed by normal and malignant cells which trigger a complex proinflammatory response characterized by release of IL-1, IL-6, IL-8, TNF-α and GM-CSF [7], [8]. Subsequently, a variety of immune cells, including T cells, natural killer (NK) cells, neutrophils, and macrophages migrate to the bladder where they become activated and produce additional proinflammatory cytokines and chemokines [9], [10]. This is characterized by the appearance of different leukocytes and cytokines in the urine of BCG-treated patients. While the specific role that various cell types and cytokines play in urothelial carcinoma treatment is not completely clear, urinary Th1 cytokines (*e.g.*, IFNγ, IL-2, and IL-12) have been associated with the BCG response, whereas high levels of Th2 cytokines (*e.g.*, IL-10 and IL-6) correlate with BCG failure [9]–[16]. Overall, the ability of intravesical BCG to interact with the urothelium and stimulate broad, durable Th1- type immune responses is probably critical to its efficacy. As a result, it has been suggested that the addition of immunostimulatory agents, such as cytokines, to BCG therapy could augment these immune responses and provide greater and/or more durable antitumor effects. However, to date, phase 3 clinical investigations of intravesical BCG in combination with IL-2 or IFN-α2b have been limited with only one trial reported, which did not demonstrate benefit over BCG monotherapy [17]. Thus, optimal strategies for maximizing the immunotherapeutic efficacy of intravesical treatment of NMIBC will likely require novel approaches.

IL-15 is a critical factor in the development, proliferation and activation of effector natural killer cells and CD8^+^ T cells [18], [19] and has been listed as the most promising agent among twelve immunotherapy drugs with a high potential for use in treating human cancers [20]. We have recently isolated a novel IL-15 mutant with a 4-fold increase in biological activity [21]. The pharmacokinetics (PK), biodistribution and biological activity of this IL-15 superagonist (IL-15N72D) have been further improved through interaction with the soluble domain of the IL-15 receptor α protein (IL-15RαSu) to create the IL-15N72D/IL-15RαSu-Fc fusion complex (referred to as ALT-803). This complex has at least 25-times the *in vivo* biologic activity of native IL-15 [22], [23]. We have recently demonstrated that systemic ALT-803 treatment induces durable and protective immune cell-mediated antitumor responses in several mouse models for hematologic malignancies [22], [23]. ALT-803 stands out as a potent immunostimulant that is capable of simultaneously activating the innate and adaptive arms of the immune system to elicit both rapid and long-lasting protective responses against infectious or neoplastic challenges to the host [24].

Motivated by the compelling pre-clinical data related to IL-15, we tested the therapeutic efficacy of intravesical ALT-803 alone and in combination with BCG in a well-established rodent carcinogen induced NMIBC model. Specifically, we observed that a) intravesical ALT-803 was safe and well tolerated alone and in combination with BCG, b) ALT-803 plus BCG reduced tumor burden by 46% compared to control, which was higher than with either agent alone, c) immune monitoring suggested that the antitumor response seen with the combination of ALT-803 plus BCG was linked to the production and secretion of IL-1α, IL-1β and RANTES, which in turn, induced the proliferation and activation of NK cells and d) tumoral responses seen with the combination of ALT-803 and BCG were associated with a significant reduction in angiogenesis. The encouraging results presented in this report will support our consideration for further development of this novel therapeutic duplet in the treatment of patients with NMIBC.

## Materials and Methods

### Animals, reagents, and tumor model

Ninety female Sprague Dawley rats, six to eight weeks old, were obtained from Harlan Laboratories (Indianapolis, IN). Animal care was in compliance with the recommendations of *The Guide for Care and Use of Laboratory Animals* (National Research Council) and approved by our local IACUC at the University of Central Florida. N-butyl-N-(4-hydroxybutyl) nitrosamine (BBN) was purchased from TCI America (Portland, OR), aliquoted and dissolved in 1% Tween. ALT-803 (IL-15N72D∶IL-15RaSu/Fc) was generated as described previously [21] and diluted with sterile phosphate buffered saline (PBS). PBS served as negative control. Lyophilized BCG was purchased from Sanofi Pasteur Limited (Toronto, CA) and reconstituted as per instructions with sterile PBS and served as positive control. After allowing animals to acclimate to our facility for one week, all animals except five received 0.05% BBN in their drinking water continuously for eight weeks inducing the formation of NMIBC, which is a well-established rodent carcinogen induced BCa model [25]–[30] with marked molecular changes similar to human BCa [31]. After 8 weeks of BBN exposure, animals resumed water without the addition of BBN.

### Intravesical treatment

**Figure 1:**
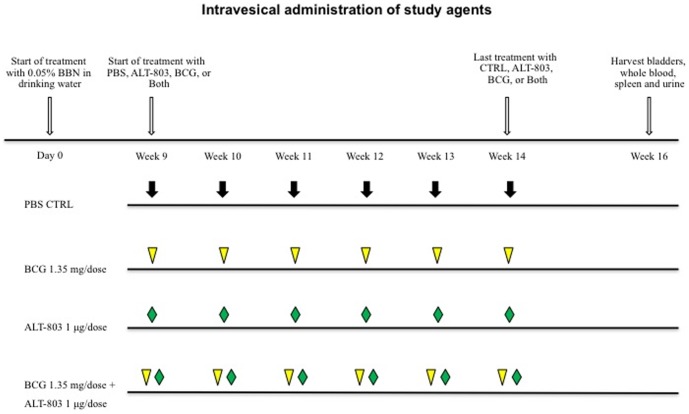
Study treatment schema. Treatment with PBS, BCG, ALT-803, or ALT-803 plus BCG commenced one week after completion of the eight weeks exposure to 0.05% N-butyl-N-(4- hydroxybutyl) nitrosamine in drinking water. Rats were randomly assigned into four groups and treated weekly for six consecutive weeks according to the schedule. Rats were sacrificed and necropsied two weeks later. CTRL, control.

After BBN exposure (week 9), rats were randomized to one of four treatment groups (control- PBS; ALT-803 at 1 µg/dose; BCG at 1.35 mg/dose [26], [27], or ALT-803 at 1 µg/dose plus BCG 1.35 mg/dose). Each group contained 20 animals. Preliminary experiments with ALT-803 noted a) therapeutic response with an intravesical dose of 1 µg and b) improved therapeutic response with the concomitant administration of ALT-803 plus BCG compared to the sequential administration of these two agents (data not shown). All bladder instillations were done in 200 µl of sterile PBS. Rats were anesthetized with isoflurane then a 22-gauge Teflon angiocatheter (transurethral catheter) was placed into the bladder via the urethra and urine was completely drained from the bladder [32]. Next, PBS, BCG, ALT-803, or combination of ALT-803 plus BCG therapy was delivered by transurethral instillation through the transurethral catheter and allowed to dwell in the bladder for 1 hour by occlusion of the urethra with a purse string suture. After 1 hour, the purse string suture was removed and rats were stimulated to expel bladder contents [32]. The intravesical therapy was administered weekly for a total of six weeks to mimic intravesical BCG therapy in humans [33]. Two weeks after completion of the intravesical therapy, animals were sacrificed and tissues (*e.g.*, whole blood, serum, urine, bladder and spleen) were harvested and processed for subsequent analysis. **Figure 1** illustrates the study treatment schema.

### Ultrasound imaging studies

Throughout the study, intravesical tumor burden was monitored *in situ* by transabdominal ultrasound imaging using the Vevo 2100 imaging system with a MS550D 22–55 MHz hand held MicroScan array transducer (Visualsonics, Toronto, ON, Canada). Briefly, rats were anesthetized with isoflurane then 200 µl of sterile PBS was instilled into the bladder as described above to provide a uniform image of all bladders visualized. Intraluminal tumors were observed and digital images captured. Bladder wall thickness (in millimeters), a surrogate of tumor burden, was measured with the Vevo 2100 measurement feature.

### Histopathology of tumor sections

Resected bladders were initially weighed then 12 bladders from each group were filled with 200 µl of 10% neutral buffered formalin. The bladder necks were ligated and the entire specimens placed in 10% neutral buffered formalin. Bladders in formalin were embedded in paraffin, sectioned (5 µm) and placed on Superfrost plus Micro slides (Fisher Scientific, Pittsburgh, PA). The remaining eight bladders in each group were filled with 200 µl of O.C.T. embedding compound (Andwin Scientific, Schaumburg, IL) and the bladder necks were ligated. The bladders were submersed in Tissue-Tek O.C.T. compound (Ted Pella, Inc., Redding, CA) in cryomolds and frozen in liquid nitrogen and stored at −80°C. Bladders frozen in O.C.T. were sectioned (5 µm), warmed at room temperature for 5 minutes, fixed in ice-cold methanol for 10 minutes, and washed with PBS prior to immunohistochemical staining. Deparaffinized and frozen sections from each rat were subjected to hematoxylin and eosin stain for histological evaluation. Additionally, deparaffinized slides were treated with 1% hydrogen peroxide in 100% methanol to block endogenous peroxidase activity followed by citric acid antigen retrieval. Slides were incubated overnight with antibodies against CD3^+^ (BD Biosciences; G4.18; mouse monoclonal antibody, dilution 1/250), CD8a^+^ (BD Biosciences; OX-8; mouse monoclonal antibody, dilution 1/10), CD161^+^ (AbDSerotec; 10/78; mouse monoclonal antibody, dilution 1/100) (detecting NK cells), CD163^+^ (AbDSerotec; ED2; mouse monoclonal antibody, dilution 1/50) (detecting macrophages), Ki-67 (Dakota North America Inc.; MIB-5; mouse monoclonal antibody, dilution 1/50), or cleaved caspase 3 (Cell Signaling Technology; 5A1E; rabbit monoclonal antibody, dilution 1/1 500). Frozen sections were incubated overnight with antibodies against CD4^+^ (BD Biosciences; OX-35; mouse monoclonal antibody, dilution 1/250) or CD31 (Santa Cruz Biotechnology; H3; mouse monoclonal antibody, dilution 1/250). Next, the deparaffinized and frozen sections were incubated with biotinylated anti-mouse or anti-rabbit IgG (H+L) antibodies at 10 µg/ml (Vector Laboratories INC., Burlingame, CA). Subsequently, sections were stained using the Ultra-Sensitive ABC Mouse IgG staining kit (EMD Millipore, Billerica, MA) or the Biotin/Streptavidin immunostaining kit (Fisher Scientific, Pittsburgh, PA). All stained sections were imaged using a Nikon Eclipse E400 light microscope (Nikon Inc., Melville, NY) with the QIClick digital CCD camera (QImaging, Surrey, BC, Canada) and the Nikon NIE-Elements Basic Research imaging software.

Detailed histopathologic analysis of the neoplastic response to intravesical therapy was performed in each bladder by two experienced, independent observers (MM and AS). Briefly, CD3^+^, CD4^+^, CD8a^+^ T-cells, NK cells and macrophages were counted in a minimum of four randomly selected fields per high power field (hpf) in each specimen. Results were scored by calculating the number of infiltrating immunostaining cells.

Furthermore, tumor angiogenesis was investigated on slides stained with anti-CD-31 monoclonal antibody and microvessel density (MVD, microvessel/mm^2^) was assessed as previously described by Weidner *et al.* [34]. Single endothelial cells or clusters of endothelial cells positive for CD-31 were considered as a vessel. Four areas with the highest microvessel concentration from each specimen were identified at 4× magnification and images were taken for quantitation of MVD at 200×. The two independent observers counted the number of microvessels in each histological field. MVD of the specimen was estimated as a mean of vessels in at least four histological fields. In addition, staining slides with the monoclonal antibody directed against Ki-67 assessed proliferation. Ki-67 immunostaining was counted in a minimum of four randomly selected fields from each treatment group at 200× magnification. Cells with questionable nuclear staining were discounted. The observers scored the results by estimating the percentage of tumor cells showing the characteristic nuclear staining (proliferative index). Lastly, staining slides with the monoclonal antibody directed against cleaved caspase-3 assessed apoptosis. Cleaved caspase-3 immunostaining was counted in a minimal of 3,000 cells from each treatment group at 200× magnification. The two observers scored the results by estimating the percentage of tumor cells showing the characteristic cellular staining (apoptotic index).

### Flow cytometric analysis of PMBC and spleen for T-cells, NK cells and Macrophages

Rat peripheral blood mononuclear cells collected from whole blood samples in EDTA tubes were stained directly by incubation with fluorescently labeled antibodies CD3^+^ (eBioscience), CD4^+^ (BD Biosciences), CD8a^+^ (eBioscience), NKG2D-positive (eBioscience) (detecting NK cells), F480-PE (eBioscience) (detecting macrophages), or isotype control antibodies (eBiosciences, San Diego, CA) for 30 minutes at room temperature, followed by red blood cell lysis using BD FACS Lysing Solution (BD Biosciences, San Jose, CA) at room temperature for 3–5 minutes. Cells were subsequently washed twice with PBS containing 1% fetal bovine serum. Stained cells were analyzed using a FACScan flow cytometer (BD Biosciences) and data were collected for 10,000 events/sample. Analysis of the data collected was performed using Cyflogic software (CyFlo LTD, Turku, Finland). In addition, single cell suspensions of the rats' spleens were generated by gently homogenizing tissue in a petri dish and then passing through a Cellector Tissue Sieve (Bellco Glass, Vineland, NJ). Red blood cells were removed from rat splenocytes with BD FACS Lysing Solution by incubation for 3–5 minutes at room temperature, followed by centrifugation at 1,200 rpm for 5 minutes. The pelleted splenocytes were washed twice with PBS containing 1% fetal bovine serum followed by direct immunofluorescence staining as described above.

### Urinary and serum inflammatory cytokine ELISA assay

Voided urine from each rat was collected in a tube on ice containing a concentrated urine stabilizer solution (2 M Tris-HCl [pH 7.6], 5% BSA, 0.1% sodium azide, and a COMPLETE mini protease inhibitor tablet (Roche Diagnostics, Indianapolis, IN)). Urine was centrifuged and supernatant was stored at −80°C. Similarly, serum was collected in tubes containing EDTA, centrifuged at 10,000×g for 15 minutes, and stored at −20°C. Urine and serum samples were subjected to profiling of 12 inflammatory cytokines using an ELISA assay (RAT Cytokine multi-analyte ELISArray, SABiosciences, San Diego, CA). Briefly, urine and serum samples were incubated in 96-well microplates coated with anti-rat primary antibodies against 12 inflammatory cytokines (IL-1α, IL-1β, IL-2, IL-4, IL-6, IL-10, IL-12, IL-13, IFN-γ, TNF-α, GM-CSF, and RANTES) and then developed with secondary antibodies conjugated with horseradish peroxidase. After adding the substrate and stop solution, a FLUOstar Optima Reader (BMG LABTECH, Ortenberg, Germany) was used to measure absorbance at 450 nm.

### Statistics

Experimental data were expressed as mean ± SEM, unless otherwise indicated. Statistical analysis was conducted using non-parametric Kraskal-Wallis test followed by post-hoc Dunnet's test for bladder weight, bladder wall thickness, immunostaining of the bladder, urinary cytokines and serum cytokines. Parametric ANOVA test followed by post-hoc Bonferroni's test was performed for the data related to lymphocytes within spleen and lymphocytes within the peripheral blood. A *p*<0.05 was considered significant. All statistical analyses and figures were carried out using GraphPad Prism software 5.0 (GraphPad Software, Inc., La Jolla, CA).

## Results

### Antitumor activity of BCG, ALT-803 and ALT-803 plus BCG in NMIBC-bearing rats

In a rodent carcinogen induced NMIBC model, routine monitoring of tumors was performed by serial ultrasound imaging with the Vevo 1200 system. Tumors were detectable as early as 3 weeks after exposure to 0.05% N-butyl-N-(4-hydroxybutyl) nitrosamine in drinking water. As demonstrated by ultrasound imaging and confirmed on histologic examination, 100% of animals were found to develop tumors. No animals had palpable, advanced tumors. To determine if ultrasound imaging could be used to monitor and assess tumor burden, bladders were imaged and bladder wall thickness (a surrogate for tumor burden reported in millimeters) was determined. Subsequently bladders were resected, weighed and histopathologically examined. We noted a positive correlation (Spearman R = 0.55, *p*<0.0001) between bladder wall thickness and bladder weights/bladder tumor, thus we report for the first time that the non-invasive *in situ* monitoring of rodent's bladder tumor may be used to a) confirm tumor take and b) report therapeutic tumor-burden reduction.

Rats bearing bladder tumors were treated intravesically for 6 consecutive weeks with PBS, BCG, ALT-803 or ALT-803 plus BCG. Other than the scant hematuria noted (perhaps from catheterization and/or BCG), intravesical therapies were well tolerated with no appreciable toxicities noted.

**Figure 2:**
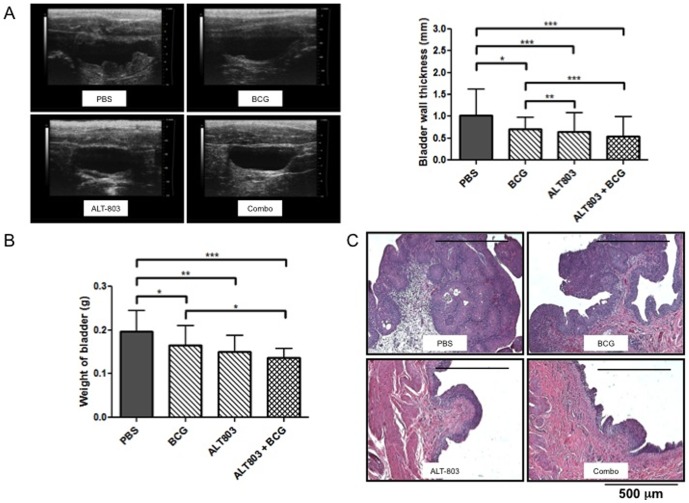
Antitumor activity of BCG, ALT-803 and ALT-803 plus BCG. NMIBC were induced in female Sprague Dawley rats by the exposure to 0.05% N-butyl-N-(4-hydroxybutyl) nitrosamine in drinking water. At week 9, rats were randomized to one of four treatment groups (PBS, BCG, ALT-803, or ALT-803 plus BCG). Each group received weekly intravesical therapy for six consecutive weeks. On week 16, rats were sacrificed. **A**) Prior to bladder resection, 200 µl of sterile PBS was instilled into bladder and imaging of the bladder was performed with the Vevo 2100 Ultrasound System from VISUALSONICS (22–55 MHz probe). Thickness of the bladder wall was recorded in millimeters. Non-parametric Kraskal-Wallis test followed by post-hoc Dunnet's test was performed. Representative images from the four groups (*top left*) along with mean bladder wall thickness (*top right*) are illustrated. **B**) Resected bladders were fixed in 10% buffered formalin and embedded in paraffin and subsequently stained with hematoxylin and eosin. Detailed histopathologic examination of each bladder was performed to assess tumor burden. Representative images from the four groups are illustrated. The greatest reduction in bladder tumor burden was seen in ALT-803 and ALT-803 plus BCG. **C**) Bladder wall thickness was reduced in groups treated with ALT-803, BCG and ALT-803 plus BCG compared to control. Greatest reduction in bladder wall thickness was seen in the group treated with ALT-803 and ALT-803 plus BCG (*p* = 0.0001). **D**) Bladder weight was reduced in groups treated with BCG alone, ALT-803 alone and ALT-803 plus BCG compared to control. Greatest reduction in bladder weight was seen in the group treated with ALT-803 plus BCG. *, *p*<0.05, **, *p*<0.01, ***, *p*<0.001.

Consistent with the development of *in situ* tumors, bladder weights increased by 49% in animals receiving BBN when compared to normal (no BBN) control animals (data not shown). However after six weeks of intravesical therapy with BCG, ALT-803 or ALT-803 plus BCG significantly decreased the tumor burden in bladders of the BBN-treated rats (**Fig. 2**). Bladder weight (mean+/−SEM) was reported to be: PBS- 0.2+/−0.012 grams, BCG- 0.17+/−0.012 grams, ALT-803- 0.15+/−0.0091 grams and ALT-803 plus BCG- 0.14+/−0.0054 grams. In addition prior to being sacrifice at week 16, 200 µl of sterile PBS was instilled into each bladder to ensure uniformity in size and a series of transverse and sagittal ultrasound images were obtained. Bladder wall thickness (mean+/−SEM) was reported to be: PBS- 1+/−0.11 mm, BCG- 0.7+/−0.05 mm, ALT-803- 0.65+/−0.077 mm and ALT-803 plus BCG- 0.54+/−0.083 mm. Thus from these images, we noted with BCG treatment a 30% reduction in mean bladder wall thickness, which corresponded to a 15% reduction in bladder weight change compared to PBS treatment. ALT-803 treatment alone was noted to have a 25% reduction in mean bladder wall thickness and a corresponding 35% reduction in bladder weight change when compared to PBS treatment. Interestingly, the greatest reduction in bladder wall thickness and bladder weight was seen when ALT-803 was combined with BCG leading to a 30% reduction in bladder wall thickness and a corresponding 46% reduction in bladder weight change compared to PBS treatment (**Fig. 2a–2c**). All results were subsequently confirmed by detailed histologic examination (**Fig. 2d**) in which H&E-stained sections were microscopically examined and classified as (a) normal urothelial mucosa characterized by epithelium of less than three layers without any dysplasia or (b) urothelial carcinoma [29].

### Intravesical ALT-803 plus BCG promotes a unique lymphocytic infiltration

**Figure 3:**
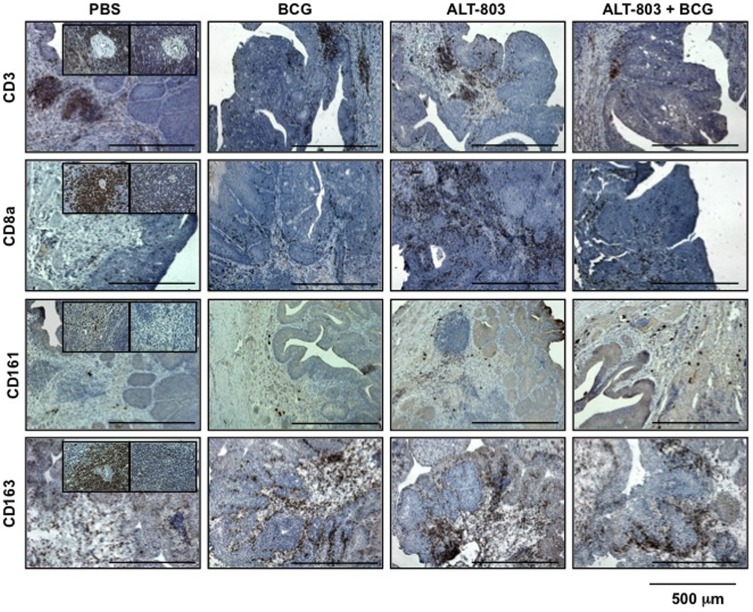
Treatment-related changes in key lymphocytic cell tumoral infiltration. Tumoral infiltration of cells positive for CD3^+^, CD8a^+^, CD161 (NK cell), or CD163 (macrophage) (200×) were noted. No changes were noted in CD4^+^ T-cells (*data not shown*). ALT-803 alone increased tumoral CD8a^+^ expressing cells, whereas ALT-803 plus BCG increased tumoral expression of NK cells (See Table S1). *Insert*, left panel positive control from rat spleen and right panel negative control from rat spleen without primary antibody.

The bladders of treated rats were also stained for immune cell (CD3^+^, CD4^+^, CD8a^+^ T-cells, NK cells and macrophages) infiltrates (**Fig. 3** and Table S1). The number of CD3^+^ T-cells was significantly increased in ALT-803 alone compared to PBS and BCG alone. Combination of ALT-803 plus BCG did not improve upon the number of infiltrating CD3^+^ T-cells compared to ALT-803 alone, but when compared to BCG alone, the number of infiltrating CD3^+^ T-cells was significantly higher in the combination group. CD4^+^ T- cells were not affected in any group compared to PBS (*data not shown*). CD8a^+^ T-cells were significantly increased in BCG alone, ALT-803 alone and ALT-803 plus BCG compared to PBS. The combination of ALT-803 and BCG did not have a larger effect than BCG alone or ALT-803 alone. Next, the number of NK cells was not increased in BCG alone or ALT-803 alone compared to PBS, but the number of NK cells was increased when ALT-803 was added to BCG. Furthermore, the combination of ALT-803 plus BCG resulted in increased infiltrating NK cells compared to BCG alone or ALT-803 alone. No significantly changes in macrophage infiltration were observed in any treatment group.

**Figure 4:**
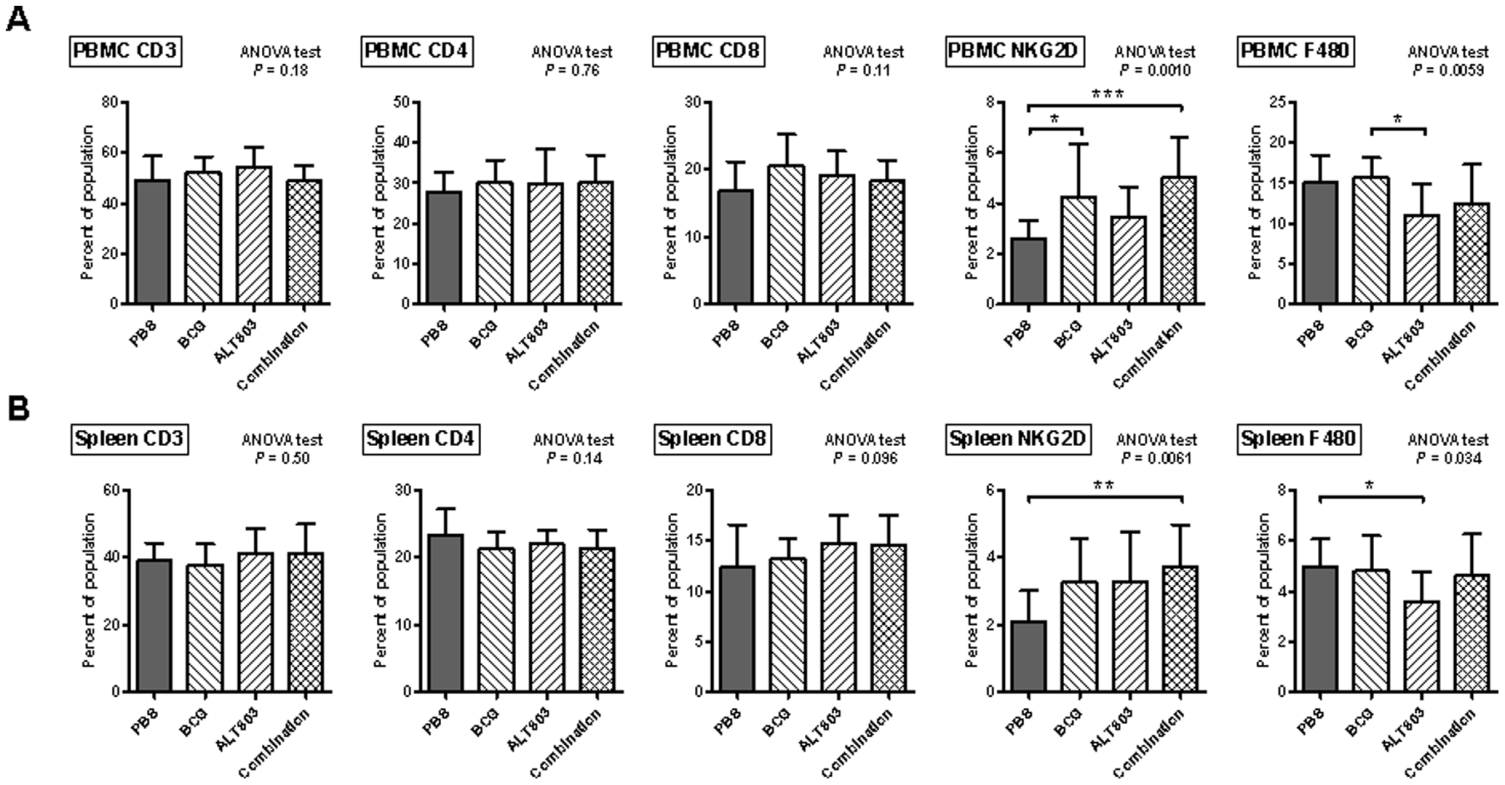
Treatment-related activation of peripheral immune cells in female rats with carcinogen induced NMIBC. **A**) Peripheral blood mononuclear cells (PBMC) were isolated and analyzed by flow cytometry for expression of CD3^+^, CD4^+^, CD8a^+^ T-cells, NKG2D- positive (NK cells) and F480 expression cells (macrophages). ALT-803 alone, as well as, ALT- 803 plus BCG resulted in an increase in NKG2D-positive cells and a decrease in macrophages compared to PBS. **B**) Splenocytes were isolated and analyzed by flow cytometry for expression of CD3^+^, CD4^+^, CD8^+^, NKG2D-positive and F480 expression cells. Similar to the PBMC results, ALT-803 alone, as well as, ALT-803 plus BCG resulted in an increase in NKG2D- positive cells and a decrease in macrophages compared to PBS. *, *p*<0.05, **, *p*<0.01, ***, *p*<0.001.

PBMC in whole blood from rats treated on this protocol was collected immediately after the animals were sacrificed and subjected to flow cytometric analysis for the presence of CD3^+^, CD4^+^, CD8a^+^ T-cells, NKG2D-positive cells and macrophages. No appreciable changes were noted for CD3^+^ and CD4^+^ T-cells, while CD8a^+^ T-cells were noted to be significantly elevated only in BCG alone group. All groups (BCG alone, ALT-803 alone and ALT-803 plus BCG) demonstrated a significant increase in NKG2D-positive cells compared to control. Lastly, the number of circulating macrophages was significantly reduced in ALT-803 alone and ALT-803 plus BCG, but not in BCG alone (**Fig. 4a**). Similar results were obtained from the analysis of the rat splenic tissues. Specifically, no appreciable changes were noted with CD3^+^, CD4^+^ and CD8a^+^ T-cells amongst the treatment groups. However, the number of NKG2D-positive cells was significantly increased in the spleens from rats in all groups (BCG, ALT-803 and ALT-803 plus BCG) compared to control. Combination of ALT-803 plus BCG did not note an increase in the number of NKG2D-positive cells compared to BCG alone or ALT-803 alone. Furthermore, the number of macrophages within the spleen was significantly reduced in ALT-803 alone when it was compared to PBS and BCG alone. The number of macrophages returned to baseline when ALT-803 was added to BCG (**Fig. 4b**). These data provide compelling evidence that the combination of ALT-803 plus BCG is associated with the stimulation of NKG2D-positive cells, which was likely the effector cell mediating tumor regression during intravesical combination immunotherapy.

### Intravesical administration of ALT-803 plus BCG facilitated unique cytokine profile

**Figure 5:**
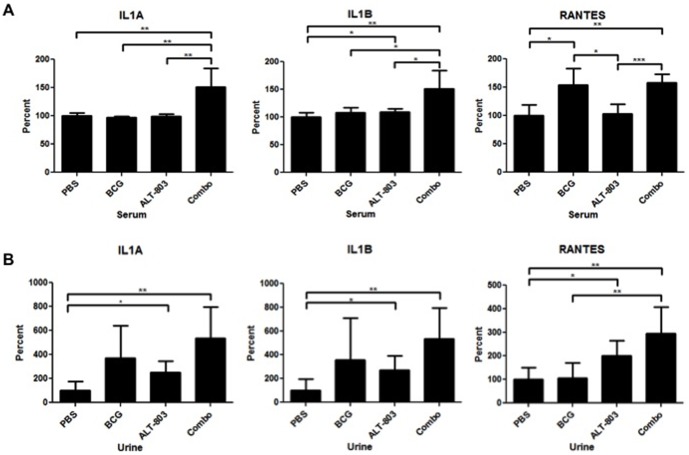
Changes in serum and urinary cytokine profiles following intravesical treatment. **A**) Serum cytokines - Serum samples from female rats with carcinogen induced NMIBC was compared among the four treatment groups. Serum levels of a panel of cytokines were measured by Rat Inflammatory Cytokines Multi-Analyte ELISArray Kit (SABiosciences, Valencia CA). Mean ± SEM of each cytokine is reported for each treatment group. **B**) Urinary cytokines - Voided urine samples from female rats with carcinogen induced NMIBC was compared among the four treatment groups. Rat Inflammatory Cytokines Multi-Analyte ELISArray Kit measured urine levels of a panel of cytokines. Mean ± SEM of each cytokine is reported for each treatment group. *, *p*<0.05, **, *p*<0.01, ***, *p*<0.001.

Sera from rats treated on this protocol were collected and immediately stored at −80°C. For the cytokine analysis, sera samples were thawed and then subjected to analysis with the rat inflammatory cytokines multi-analyte ELISArray assay. Along with proliferation and activation of NK cells noted above, the intravesical administration of ALT-803 in combination with BCG was associated with increased serum levels of the inflammatory and immune response cytokines, IL-1α and IL-1β by 52% and 52%, respectively, compared to that of the PBS control group. Furthermore, the increase in IL-1α and IL-1β was maintained when ALT-803 plus BCG was compared to BCG alone and ALT-803 alone (**Fig. 5a**). Next, urine from rats treated on this protocol were collected and stored at −80°C immediately after the animals were sacrificed. When ready for analysis, urine samples were thawed and subjected to the rat inflammatory cytokines multi-analyte ELISArray assay. Once again, the cytokines, IL-1α, IL-1β and RANTES were increased by 437%, 437% and 196%, respectively, in the urine of the ALT-803 plus BCG treated rats compared to the PBS control group. Nevertheless, BCG alone and ALT-803 alone were associated with increases in IL-1α, IL-1β. Only RANTES was significantly elevated in ALT-803 plus BCG when compared to BCG alone and ALT-803 alone (**Fig. 5b**). Within the serum, IL-1α and IL-1β are key cytokines, while locally RANTES is the key cytokine.

### Comparison of bladder tumor proliferation, apoptosis and angiogenesis following intravesical therapy

**Figure 6:**
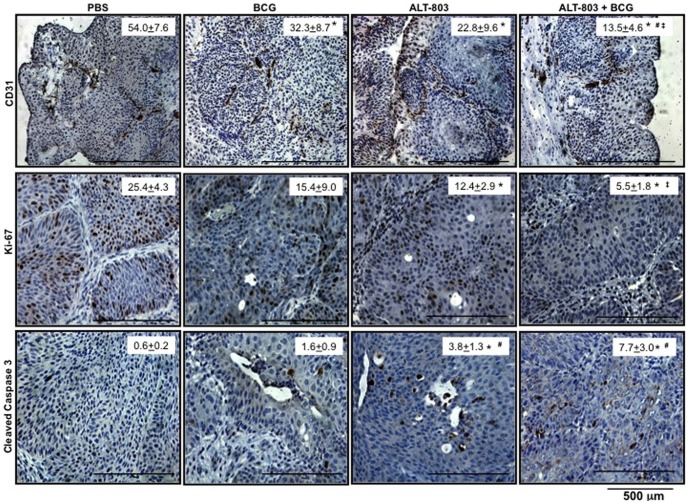
Treatment-related changes in key biomarkers in bladders of female rats with carcinogen induced NMIBC. Analysis of bladder angiogenesis (MVD), proliferation index (PI) and apoptosis index (AI) by IHC (200×) as described in the Material and Methods. ^*^, *p*<compared to PBS; ^#^, *p*<0.05 compared to BCG, **^‡^**, *p*<0.05 compared to ALT-803.

We believe the primary mode of action for ALT-803 and BCG is the result of this duplet's ability to stimulate the secretion of IL-1α, IL-1β and RANTES, which led to the activation and infiltration of NK cells. However, we also sought to determine if these cytokines affected key tumoral processes (*e.g.*, angiogenesis, proliferation and apoptosis) (**Fig. 6**). First, we set out to assess the angiogenic potential within each group by reporting the MVD. MVD was significantly reduced by 40% in the BCG alone group, 59% in the ALT-803 alone group and by 76% in the ALT-803 plus BCG group. Next, counting the number of Ki-67 expressing cells per hpf assessed the proliferative index. Compared to control, we noted a 40% reduction of the proliferative index in the BCG alone group, 52% reduction in the ALT-803 alone group and 80% reduction in the ALT-803 plus BCG group. Finally, the number of cleaved caspase-3 positive cells per hpf was measured to derive an apoptotic index. Compared to control, apoptosis was increased by 1.6× in the BCG alone group, by 3.8× in the ALT-803 alone group and by 7.7× in the ALT-803 plus BCG group.

## Discussion

As previously described, the IL-15N72D mutein was generated through site-directed mutagenesis of IL-15 wild type and found to exhibit super agonist activity through improved interactions with the human IL-15Rβ chain [21]. The IL-15N72D mutein displayed ∼4 fold lower effective concentration (EC_50)_ than wild-type IL-15 and the super agonist activity was enhanced further by pre-association with either mouse or human IL-15Rα-Fc molecules [21], [22]. Overall, the IL-15N72D/IL-15Rα-Fc (ALT-803) complex exhibited a 25-fold lower EC_50_ than IL-15 alone for supporting IL-15-dependent cell growth, indicating the complex provides highly potent biological activity. The enhanced activity of the IL-15N72D/IL-15Rα-Fc complex is likely the result of a combination of the increased binding activity of the N72D mutein to the IL-2Rβγ complex, optimized cytokine trans-presentation by the IL-15Rα chain, the dimeric nature of the cytokine domain and its increased *in vivo* half-life compared to native IL-15 [35], [36]. Recently, Xu *et al.* reported on the therapeutic efficacy of intravenous ALT-803 in a myeloma xenograft model and alluded to the possible mechanism behind ALT-803 activity [23]. Briefly, a single dose of ALT-803, but not IL-15 alone, eliminated well-established 5T33P and MOPC-315P myeloma cells from the bone marrow of tumor-bearing mice and prolonged survival in myeloma-bearing mice. ALT-803 treatment stimulated CD8 cells to secrete IFN-γ and promoted rapid expansion of CD8(+)CD44(high) memory T-cells. These memory CD8(+) T- cells also exhibited nonspecific cytotoxicity against myeloma and other tumor cells *in vitro*, whereas IFN-γ had no direct effect on myeloma cell growth *in vitro*. Though ALT-803 lost its antimyeloma activity in tumor-bearing IFN-γ knockout mice, it was able to retain its ability to promote CD8(+)CD44(high) memory T-cell proliferation, indicating that ALT-803-mediated stimulation of CD8(+)CD44(high) memory T-cells is IFN-γ-independent [23]. ALT-803 stands out as a potent immunostimulant that is capable of simultaneously activating the innate and adaptive arms of the immune system to elicit both rapid and long-lasting protective responses against infectious or neoplastic challenges to the host [24]. Thus, we hypothesize that intravesical administration of ALT-803 plus BCG will provide durable and potent cell-mediated immune responses, which would result in a highly efficacious and potentially curative immunotherapy for patients with NMIBC.

In the current study, we confirmed that ALT-803 stimulated CD8^+^ T-cells proliferation. Unlike in the study by Xu *et al.* [23], we did not note an increase in IFN-γ levels associated with the proliferation of CD8a^+^ T-cells, but this could be due to the fact that we did not assess cytokine levels until two weeks after the last dose of ALT-803 was administered. Thus, IFN-γ production may be a more immediate response to IL-15 stimulation. The novelty of our study is that the combination of ALT-803 plus BCG stimulated the production of key sera and urinary cytokines (*e.g.*, IL-1α, IL-1β and RANTES) leading to the activation and proliferation of NK cells, which led to the significant reduction in tumor burden. Interestingly, ALT-803 alone resulted in similar tumor reduction and production of urinary IL-1α, IL-1β and RANTES, which also led to the activation and proliferation of CD8a+ T-cells. However, the reduction in angiogenesis was significantly enhanced when ALT-803 was combined with BCG compared to ALT-803 alone. Specifically, tumoral responses to the combinational therapy were associated with 76% reduction in angiogenesis compared to only a 40% and 59% reduction in angiogenesis in BCG alone and ALT-803 alone groups, respectively. Furthermore, there was a trend that ALT-803 plus BCG resulted in reduced rates of cellular proliferation and increased rates of apoptosis compared to PBS or either agent alone. Thus, we believe if tumors were assessed at later time points that a greater reduction in tumor burden may be evident in ALT-803 plus BCG compared to ALT-803 alone. Our results provide strong evidence for ALT-803 as a viable and promising therapeutic agent against NMIBC. Herein, we present significant findings for the antitumor activity of ALT-803 in combination with the standard therapeutic modality for BCa treatment, BCG. Our results show representative reductions in tumor burden. Additionally, ALT-803 plus BCG therapy was noted to stimulate the production and secretion of key cytokines, IL-1α, IL-1β and RANTES, which in turn, induced the proliferation and activation of NK cells.

Previous studies have reported that IL-15 can induce CD8+ T-cells [23], [37], NK cells [38], and macrophages [39]. Using ALT-803, we were also able to demonstrate an induction in CD8a^+^ T-cells, which also occurred with the administration of BCG. The activation of CD8+ T- cells by intravesical BCG administration has been previously reported [40]. We observed the activation and proliferation of NK cells in the peripheral blood and spleen of animals treated with ALT-803 plus BCG. This was associated with an antitumor response and a marked increase in infiltrating NK cells within the bladder along with secretion of factors involved in inflammatory and immune responses. Among the locally elevated immune factors triggered by the combination of ALT-803 and BCG treatment within the bladder included IL-1α and IL-1β, which are both secreted by activated macrophages and neutrophils and are known to play major roles in the initiation and regulation of inflammation [41]. BCG has previously been linked to the up-regulation of IL-1α and IL-1β [42]. More importantly, RANTES was significantly increased in both the serum and urine in rats treated with ALT-803 plus BCG. RANTES is expressed and secreted by T-cells and promotes antitumor immunity by recruitment and activation of NK cells [43] and has been linked to IL-15 [44]. These induced immune responses observed following the intravesical treatment with ALT-803 plus BCG regimen were consistent with the observed effects of IL-15 on the host immune system when IL-15 was administrated intravenously [45].

Adjuvant intravesical BCG treatment has proven to be superior to other agents in the management of NMIBC and is the most commonly prescribed therapeutic agent for this population [46]. In addition, the use of maintenance intravesical BCG after induction BCG therapy was previously shown to be superior compared to no maintenance BCG [47]. In fact, current guidelines recommend the use of induction and maintenance BCG in patients with intermediate and high risk NMIBC [2]–[4]. However, although BCG treatment has been shown to delay tumor progression and improve overall survival, its efficacious is limited to early stage disease and up to 50–60% of patients fail BCG therapy and recur [5]. BCG is a nonspecific immune stimulant that evokes a complex immune response in the bladder, beginning with its binding to fibronectin and integrin-mediated internalization by urothelial cells followed by expression of various pro-inflammatory cytokines [48]. This, in turn, induces recruitment and activation of immune effector cells resulting in improved recognition and killing of the tumor by both non-specific and specific mechanisms.

Recent studies suggest IL-15 may stimulate effective antitumor responses in a variety of cancers, including BCa [49]–[51]. In one study by Matsumoto *et al.*, IL-15 gene therapy resulted in a marked inflammatory response with infiltration of CD8^+^ T-cells in the vicinity of mouse bladder tumors. The investigators also confirmed an inhibition in growth of a subcutaneous MBT-2 murine BCa cells as a result of tumor-specific cytotoxic T lymphocyte recruitment [51]. Obviously gene therapy has significant limitations, which may prohibit it from successfully advancing into clinical trial [52], whereas ALT-803 has a negligible toxicity thus making it more likely to move towards an early phase clinical trial.

No new agent has been FDA approved for the treatment of NMIBC since 1998 [53]. Better therapeutic alternatives are desperately needed for patients suffering from NMIBC. With its ability to activate NK cell responses and support T memory cell proliferation, IL-15-based therapies would be expected to further augment the antitumor activity of BCG, consistent with the results with ALT-803 plus BGC shown in this study. Indeed, improved clinical therapies may rely on the intravesical combination of traditional agents (*i.e.*, BCG) with novel compounds (*i.e.*, ALT-803).

## Conclusions

While BCa may be initially sensitive to chemotherapy and BCG, recurrence may develop and thus current regimens have resulted in only an incremental gain in treatment success. Thus there is universal agreement that better therapeutic strategies are needed for patients with NMIBC to improve disease specific survival and obviate the need for radical extirpative surgery such as cystectomy. In this study, we have clearly demonstrated the therapeutic activity of ALT-803 plus BCG in NMIBC. Specifically, we found ALT-803 plus BCG to be superior to intravesical BCG alone or ALT-803 alone not only in terms of tumor burden reduction, but also in local cytokine production and secretion, as well as activation and proliferation of NK cells. As a result, clinical investigation of intravesical ALT-803 plus BCG as a treatment regimen for NMIBC is highly warranted.

## Supporting Information

Table S1 **Comparison of mean (+SD) number of immunostaining cells infiltrating rats' bladders in each treatment group.**
(XLSX)
